# Revisiting kinetic boundary conditions at the surface of fuel droplet hydrocarbons: An atomistic computational fluid dynamics simulation

**DOI:** 10.1038/srep25572

**Published:** 2016-05-24

**Authors:** Rasoul Nasiri

**Affiliations:** 1 Sir Harry Ricardo Laboratories, Advanced Engineering Centre, School of Computing, Engineering and Mathematics, College of Life, Health and Physical Sciences, University of Brighton, Cockcroft Building, Lewes Road, Brighton BN2 4GJ, United Kingdom

## Abstract

The role of boundary conditions at the interface for both Boltzmann equation and the set of Navier-Stokes equations have been suggested to be important for studying of multiphase flows such as evaporation/condensation process which doesn’t always obey the equilibrium conditions. Here we present aspects of transition-state theory (TST) alongside with kinetic gas theory (KGT) relevant to the study of quasi-equilibrium interfacial phenomena and the equilibrium gas phase processes, respectively. A two-state mathematical model for long-chain hydrocarbons which have multi-structural specifications is introduced to clarify how kinetics and thermodynamics affect evaporation/condensation process at the surface of fuel droplet, liquid and gas phases and then show how experimental observations for a number of *n*-alkane may be reproduced using a hybrid framework TST and KGT with physically reasonable parameters controlling the interface, gas and liquid phases. The importance of internal activation dynamics at the surface of *n*-alkane droplets is established during the evaporation/condensation process.

Understanding of interfacial phenomena has become crucial to design the wide range of materials namely surfactants^1^, polymers and biopolymers^2^, electro-catalysis^3^ and other important ones^4^, but it is still far from being completely understood. Physics and chemistry of associated bonds between atoms and molecules at the interface turn out to be rather different in comparison with liquid and gas phases due to anisotropic effects in Gibbs free energy^5^. Many theoretical^6^,^7^,^8^ and experimental^9^ techniques have been developed for studying of liquid-vapour phase transitions.

Indeed, atomic level simulations using the empirical force fields (FF) which are parameterized by fixed charge scheme and/or non-self-consistent electronic structure methods are not always reliable. The reliability of the FF approach becomes particularly questionable for molecules with multiple conformers especially at the interface and extreme conditions such as internal combustion engine-like conditions (e.g., high temperatures and pressures). This deficiency in FFs in such a situation is related to the fact that the internal rotations or torsions of long chain molecules are parameterized using FFs based on a single conformer, while characterizations of these sorts of internal molecular dynamics in the hydrocarbon molecules, the main components of fuel droplets, with multi-structural effects change from one another^10^. This is supported by the results obtained for orientation of *n*-alkanes at the centre of interfacial layer using non-reactive MD simulations and experimental results^11^,^12^,^13^. Using the vibrational sum frequency spectroscopy (VSFS), which is widely applied to determine molecular orientations at the interfaces, it can be shown that the chain of *n*-alkane molecules from *n*-nonane (C_9_H_20_) to *n*-hexadecane (C_16_H_34_) are perpendicular to the surface^13^ at the temperatures well above the melting points. On the other hand, the MD simulation results using the OPLS and NERD force fields provided two different results. While in one study, *n*-dodecane molecules had mostly orientation parallel to the surface^11^, in^13^ the authors claim that *n*-decane (C_10_H_22_), *n*-tetracosane (C_24_H_50_) and *n*-hexatriacontane (C_36_H_74_) molecules have random orientation (both parallel and normal to surface) at the interface. While the FF predicted that all-trans conformer of *n*-hexadecane are dominated in the liquid phase over the other conformers^14^, quantum mechanical solvation results shown that *n*-dodecane with all-trans conformer cannot exist in the liquid phase^10^.

A kinetic boundary condition (KBC) for the Boltzmann equation can be formulated in a physically correct form if the accurate values of the evaporation or condensation coefficient are determined which have not yet been achieved for all materials^15^. The accurate calculation of this coefficient is challenging similar to the rate and equilibrium coefficients because of their exponential dependence on Gibbs free energy differences (e.g., an error few kcal mol^−1^ in estimation of overall Gibbs free energy or the Gibbs free energy of activation causes orders of magnitude in calculated rate and equilibrium constants)^16^.

The aim of this work is to introduce a mathematical model based on a hybrid method transition state theory (TST) and kinetic gas theory (KGT) to study the multiphase flows and sprays in which TST and KGT are respectively applied for better understanding of internal activation dynamics of long chain hydrocarbons at the interfacial layers and for modelling the collision effects among surrounding gases, vapour conformers, clusters and droplets at the equilibrium condition during the evaporation/condensation process. The role of thermodynamics and kinetics in determination of evaporation rate of *n*-alkanes, the main components of fuel droplets, is clarified. Despite the complexity of the processes in the gas phase, this work seeks to unravel whether non-equilibrium processes in the gas phase necessarily imply shortcomings of conventional kinetic gas theory in the sense that evaporation rate cannot be accurately determined even if interfacial phenomena are captured using TST. A critical part of this inquiry is to clarify whether KGT along with TST is suitable for reproducing experimentally observed kinetics in *n*-heptane, *n*-nonane, *n*-decane and *n*-dodecane molecules using physically reasonable parameters.

## Results and Discussion

**Figure 1:**
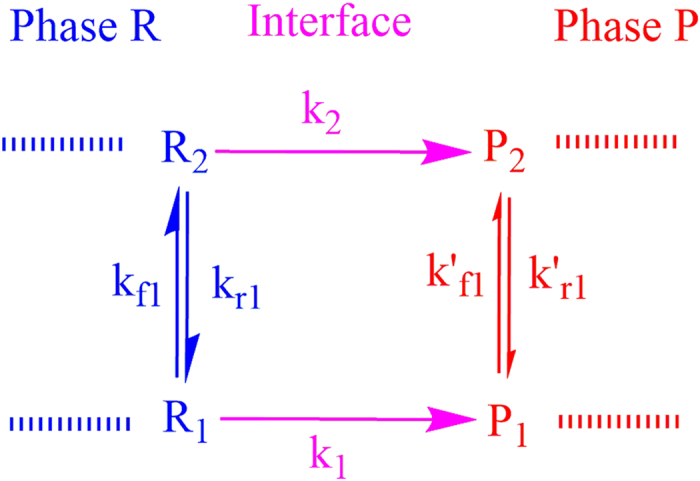
A two-state kinetic model for illustrating the evaporation/condensation kinetic effects based on the transition-state theory (TST) and kinetic gas theory (KGT) for the two conformers depicted by R_i_ and P_i_ in two **phases R and P** which are in the equilibrium state to each other and in the quasi-equilibrium state with some transition states in the interfacial layer before any transformation can take place.

**Figure 2:**
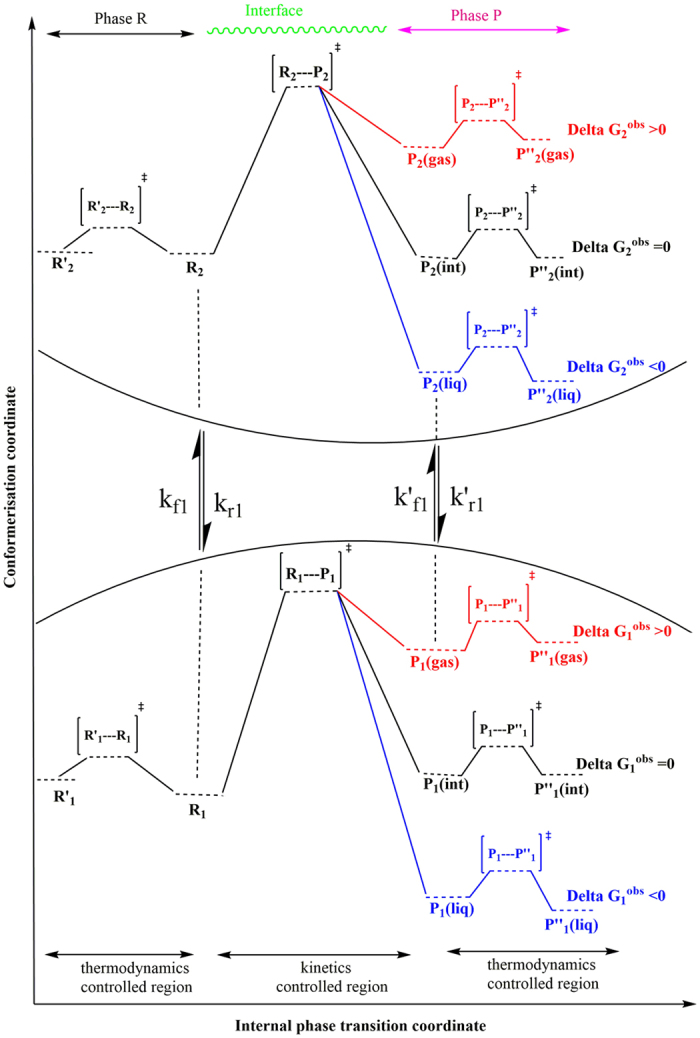
A schematic two-dimensional Gibbs free energy diagram of internal activation and conformerisation dynamics for a two-state kinetics model which occurs in two different **phases R** and **P**. The equilibrium state between conformers 1 and 2 takes place so faster than transformation of the conformers R_1_ and R_2_ to another phase (conversion R_i_ to P_i_) showing that evaporation/condensation is controlled by kinetics of internal activation process.

Here we discuss a two-state kinetic model, schematically represented in the Figs 1 and 2, applicable for the evaporation/condensation of *n*-alkane hydrocarbons in the internal combustion engine conditions.

In the Fig. 1, the reactants (R_1_ and R_2_) and products (P_1_ and P_2_) are presented as conformers which are respectively in the relevant phases R and P. When transformation of R_1_ to the phase P is an endergonic process, phase R is considered to be liquid (see red colour diagram in the Fig. 2; P_**i**_(gas)), but if the R_1_ is transferred to the phase P through an exergonic process (see blue diagram in the Fig. 2; P_**i**_(liq)), one will be dealt with condensation. Finally, a non-thermal process might occur if this conformer rests at the interfacial layer (see black diagram in the Fig. 2; P_**i**_(int)). These three possibilities take place through passing the internal activation process. While many conformers are in the equilibrium to each other in the gas phase or liquid, a limited number of conformers in the interfacial layer which are in the quasi-equilibrium with the “specific transition states” (presented as [R_i_—P_i_]^#^ in Fig. 2), might be transferred between two phases R and P. Although there may be a high number of the *n*-alkane conformers in the gas and liquid phases, the conformational changes take easily place without requiring the significant changes in the Gibbs free energy. Moreover, the experimental data such as Gibbs free energy of the aforementioned hydrocarbons in the liquid and gas phases^17^ will be fed to this mathematical model for making sure that these phases have been taken into account properly for studying the phase transitions. It is also found that these varieties in the conformers are confined to the narrow numbers of conformers in the interfacial layer because of the nano-confinement effects^18^,^19^. Since thermodynamics alone does not tell us how long these phase transitions will take to occur between two phases R and P, the phase transitions can be captured by means kinetics because of transient phenomena at the liquid-gas bridge. On the other hand, the local equilibrium between reactants (R_1_ and R_2_) and products (P_1_ and P_2_) are controlled by thermodynamics and in order to show the importance of internal molecular dynamics (IMD) effects in the evaporation or condensation of highly flexible molecules, we apply molecular theory of solvation to reveal the role of driving force of IMD (see Methods).

The hydrocarbon conformers in both phases are found to be in the quasi-equilibrium with “some transition states” leading to conformational changes with very low energy barriers (see Fig. 2). On the other hand, the same conformers are in the quasi-equilibrium states with “other transition states” at the droplet surface which have significant barrier energies leading to activation of the phase transition processes or internal activation dynamics at the interface which is controlled by kinetic effects. The rate coefficients *k*_*f*_ (or 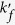) and *k*_*r*_ (or 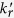) describe forward and reverse inter-conversions between the equilibrated conformers R_1_ and R_2_ (P_1_ and P_2_) with equilibrium constants 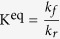 in the phase R or 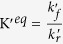 in the phase P, whereas k_1_ and k_2_ describe the respective rates for the transformation of the conformers between two phases via internal activation dynamics.

Based on the assumption that equilibration of conformers R_1_ (or P_1_) with R_2_ (or P_2_) in phase R (or P) occurs faster than internal activation processes in the interfacial layer these conformers may be in the quasi-equilibrium with their transition states. The values of internal activation energies are about one order magnitude higher than collision energies in the liquid or gas phase (~2RT_liq_ or ~2RT_gas_) and those are unlikely to be achieved at the interface. The required energies for the phase transition might be easily accessible by collision of gaseous/vapour molecules or clusters/droplets with the conformers at the surface of droplet for which three scenarios can take place; (a) scattering from the surface, (b) diffusion into the liquid phase and, (c) accommodation on the interfacial layer. The value of ΔG^obs^, as shown in the Fig. 2, determines which one will be dominated. <Δ*G*^*obs*^> is determined based on a concurrent process from two different phases for which the internal activation dynamics followed to the diffusion of the surface conformers via the interfacial layer to the gas or liquid phase when the conformers have enough energies.

This simple two–state kinetic model can be easily extended to an *n*-dimensional model in which reactant 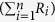 in the phase R, transition states 
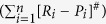
 in the interfacial layer, products 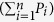 in the phase P, gaseous/vapour molecules 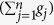 and droplets/clusters 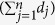 are involved during the evaporation/condensation process.

On the basis of Fig. 1, the concentration conformers R_1_ and R_2_ can be expressed as follow:


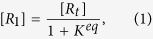



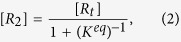


The rate for transfer of conformers from phase R to the interface can then be combined to yield:


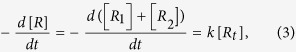


where k is the rate constant for transition of the conformers between the phase R and phase P and can be re-written as the following expression;


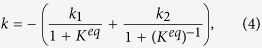


where equilibrium constant *K*^*eq*^ is equal to 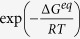 in which 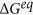 is defined as the differences Gibbs free energies between R_1_ and R_2_ conformers which are in the phase R. Therefore, equation (4) may be rewritten for the conformers as:


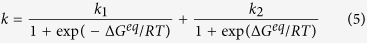


where _1_ and _2_ refer to the number of hydrocarbons conformers studied in this paper (see Table 1) which are involved during the evaporation/condensation process in the liquid phase. *k* is defined as an evaporation rate in which conformers R_1_ and R_2_ in the liquid-gas bridge are in the quasi-equilibrium state with their transition state at the interfacial layer (see Figs 1 and 2). It’s worth mentioning here the difference between Gibbs free energies of molecules in the liquid (Δ*G*^*eq*^(liq)) and the gas phases (Δ*G*^*eq*^(gas)) (hereafter presented 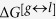) is not equal to 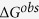 because of the kinetic effects observed at the interfacial layer such as internal activation dynamics.

In order to take into account the effects of collision among surrounding gases, vapour molecules, clusters and droplets, the collision cross-sections (CCSs) rate, Ω_*jk*_, of j^th^ gaseous/vapour conformer and clusters/droplets with the surface of k^th^ gaseous/vapour conformer and the cluster/droplet is estimated based on KGT;


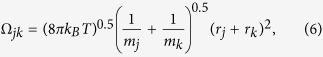


where m_j_ presents the mass of gas/vapour molecules or clusters/droplets colliding with the conformers at the surface of other clusters/droplets and gas/vapour molecules with the masses m_k_. As mentioned earlier, this collision energy will determine whether conformers R_1_ and R_2_ at the surface of the droplet can be evaporated/condensed or will be rested at the interface depends on the value of 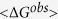 (see Fig. 2). The *r*_*j*_ is the radii of gas/vapour molecules 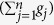 or clusters/droplets 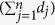 colliding with the other cluster/droplets or gas/vapour molecules 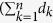 or 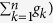 with radii of *r*_*k*_.

In order to taken into account the internal activation dynamics and the collision effects on the evaporation rate, the k_1_ and k_2_ (see equation (5)), a TST-based expression 
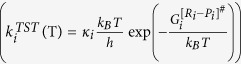
 is incorporated in the KGT-based equation 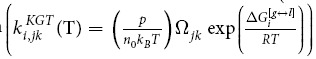 as;


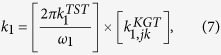



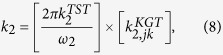


where ω_1_ or ω_2_ is the magnitude of the transition-state imaginary frequency. Despite the complexity in multiphase flow dynamics including the evaporation/condensation of fuel droplet components, this work seeks to clarify whether adding the interfacial effects using TST as a correction term to the KGT, is suitable so long as we account for evaporation of hydrocarbons with multi-structural effects. A critical part of this inquiry is to determine if this hybrid model is capable of reproducing experimentally observed kinetics in *n*-heptane, *n*-nonane, *n*-decane and *n*-dodecane droplets using physically reasonable parameters. This novel model is applying to unravel kinetic effects at the surface of droplet and make clear role of equilibrium thermodynamic in the liquid and gas phases during the evaporation/condensation process. The expression (7–8) is elaborated as the following equations for conformers 1 and 2:


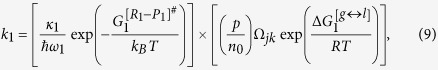



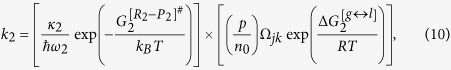


where first and second brackets are respectively expressed based on TST and KGT. It’s assumed that conformers R_1_ and R_2_ at the interfacial layer are respectively in the quasi-equilibrium with transition states [R_1_—P_1_]^#^ and [R_2_—P_2_]^#^ with having imaginary frequency of *ω*_*1*_and *ω*_*2*_ (see Figs 1 and 2). On the other hand, as shown in the second bracket, the gas-vapour mixture colliding with the surface of droplet is assumed to be in the equilibrium with molecules at the interface. In the equations (9, 
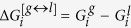
 where 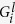 and 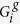 represent the values of Gibbs free energy of conformers in the liquid and gas phases, respectively. 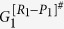 or 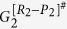 is the internal activation Gibbs free energy in the interfacial layer including zero-point energy. κ_1_ and κ_1_ are transmission coefficients including re-crossing corrections which are the changes in the conformer state in the interfacial layer during transfer into another phase via transition (internal activation) state and can be approximated as^20^:


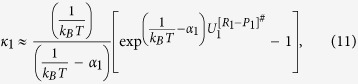



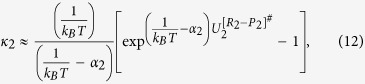


where 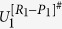 or 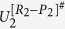 is the classical transition-state energy, which does not include zero-point energy and *α*_*1*_and *α*_*2*_ are expressed in terms of *ω*_*1*_and *ω*_*2*_, which are the magnitude of the transition-state imaginary frequency, with *α*_*i*_ = 1/(*ħω*_*i*_).

We used equation (5) to fit experimental evaporation rate for *n*-nonane, *n*-decane and *n*-dodecane droplets reported by Honnery and co-workers^21^; for *n*-heptane hydrocarbon droplet reported by Ghassemi and co-workers^22^. We constrained 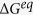 in the liquid phase^17^ to lie between −0.25 to −7.5 KJmol^−1^ for *n*-heptane, −0.24 to −7.86 KJmol^−1^ for *n*-nonane, −1.03 to −9.70 KJmol^−1^ for *n*-decane and −4.24 to −12.40 KJmol^−1^ for *n*-dodecane at the temperature range 400–700 K, so that two hydrocarbon conformers **1** and **2** represent reasonably the Gibbs free energy changes. The values of 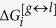 were also bounded to the experimental Gibbs free energies of evaporation^17^ in the same range of the temperatures. The experimental data obtained using the hydrocarbons density in the liquid (*ρ*_*l*_) and the gas phase (*ρ*_*g*_):


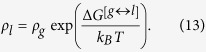


**Figure 3:**
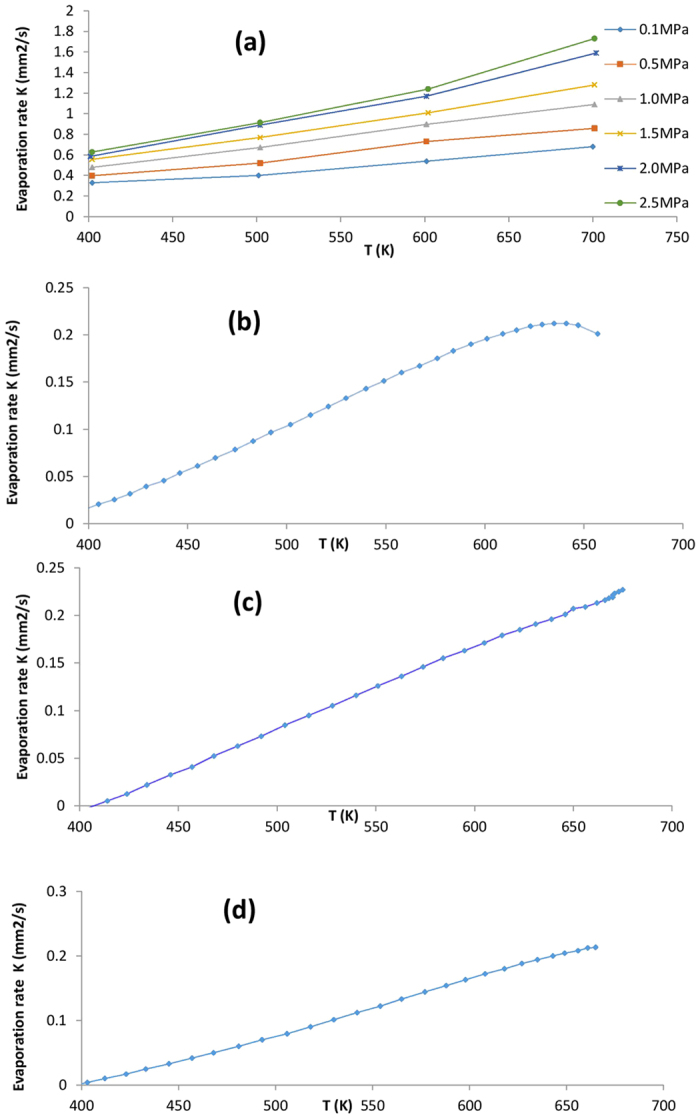
Fits to the experimental data using a hybrid model KGT and TST. The fits show that the hybrid model TST-KGT reproduces the temperature-dependent evaporation rate in the different *n*-alkane droplet. The (un)circles and solid lines respectively represent experimental measurements and the results obtained by our model — with the parameters given in Table 1. The fitted data include the droplets of *n*-alkane molecules and their initial diameters: (**a**) *n*-heptane with 1.2 mm where experimental data are available at six different pressures of 0.1 to 2.5 MPa; (**b**) *n*-nonane with 63 μm; (**c**) *n*-decane with 75 μm; (**d**) *n*-dodecane with 75 μm.

The accessible surface area of droplets during the evaporation are constrained based on the experimental results^21^ to change from 0.016596 to 0.000745 mm^2^ for *n*-decane, 0.012463 to 0.000624 mm^2^ for *n*-nonane, 0.017663 to 0.000408 mm^2^ for *n*-dodecane at 1.0 MPa gas pressure. In another experiment^22^ the droplets surface of *n*-heptane were determined to be from 5.181 to 0.471 mm^2^ at pressure 2.0 MPa. Since the zero-point effects generally lead to a larger barrier height for *n*-dodecane transfer than for *n*-heptane transfer, we constrained its transition-state frequency, to lie between 100 and 1,100 cm^−1^, which are IMD characteristics of C-C-C-C, H-C-C-H and H-C-C-C we might expect for. With these constraints at the temperature range 400–700 K, we obtained the parameters given in Table 1 and the results in Fig. 3 which show that as long as IMD is confined by the interfacial phenomena due to the nano-confinement effects, a hybrid TST- and KGT-based model is able to describe the experimental kinetic data (Fig. 3).

## Conclusions

Transition-state theory (TST) and kinetic gas theory (KGT) provide a robust and general framework for understanding evaporation/condensation of long chain molecules so long as conformational effects are taken into account, as indicated by the findings here and the hybrid model we present. This mathematical model described above reconciles the common observation of internal activation dynamic effects with TST. Our simple model uses physically realistic parameters, and is based on the concept that conformerisation and collision phenomena may be involved in evaporation/condensation processes in three different phases: here, two conformations with different internal activation dynamics and collision behaviour are sufficient to account for experimentally observed kinetic effects — in particular the unusual temperature dependence of evaporation rate in a set of *n*-alkanes. It does not invoke any new theoretical frameworks and would seem to favour the approach of our model. A full description of evaporation/condensation of fuel droplets must certainly take into account internal molecular dynamics and collision effects not only in gas and liquid phases but also in the interfacial layer. Identifying specific conformations and their internal activation dynamics roles at the vicinity of fuel droplet surface suggest nano-confinement effects in this region during the evaporation/condensation process and should be useful in better understanding transient phase transition processes in multiphase flows.

## Methods

### Molecular theory of solvation that accounts for internal molecular dynamics (IMD) effects in evaporation

We assume that liquid and gas phases are in the equilibrium at the same temperature and pressure. Note that the selection of ensample in this framework is arbitrary and it can be canonical (N, V, T) and one can deal with the Helmholtz free energy (F) rather than the Gibbs free energy (G). In order to show analytically how IMD affects the Gibbs free energy of evaporation of the hydrocarbons, N, P, T ensemble is applied to predict the Gibbs free energy of evaporation (ΔG_ev_) using molecular theory of solutions. The Gibbs free energy and chemical potential (μ) can also be considered as the same functions since G shows direction of changing of μ. We transfer a hydrocarbon molecule (C) from a fixed position respect to the centre of mass (COM) in the liquid phase into a fixed position in the ideal gas phase. Temperature T, pressure P, and the composition N (number of conformers) of the system are used here as mentioned earlier. In the traditional definitions of evaporation, one needs to specify a *standard state* in both gaseous and liquid phases for expression of;


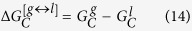


**Figure 4:**
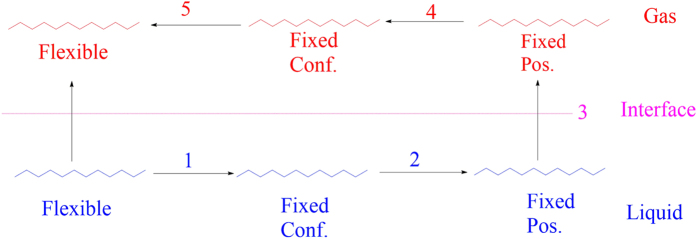
The five-step process of transferring C molecule from the liquid to the gaseous phase (conf. = conformation, pos. = position).

In our definition, there is no need to specify any standard state for the evaporation of C. This is quite clear from the definition of the above evaporation process (*vide infra*). The 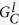 and 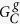 are expressed by standard definition of statistical mechanics theory as follows^23^;


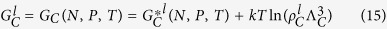



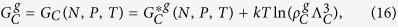


where the first terms in the right hand sides represent internal contributions of the hydrocarbon molecules and the second terms show the translational ones. Λ_*C*_ is the partition function of translational degrees of freedom which can be obtained from the integration over the distribution of momenta. The translational partition function in the gas phase becomes the liberation free energy in the solution and those are cancelled out showing that the translational partition function depends on temperature and is not affected by the interactions of the C molecule with the rest of the system. Therefore, both translational modes in the gas and liquid phases should not have any significant effects in 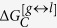 leading to the expression of 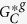
–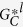 which has been defined as the Gibbs free energy of evaporation of fuel droplet hydrocarbons. Other partition functions associated with the IMD are considered in the functions of 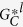 or 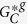 in the liquid and gas phases. The process of removing of one C from the system is performed in *five* steps as shown in Fig. 4;

Firstly, we place the C molecule at a fixed position in the liquid converting all conformers into one specific structure (all Trans conformer). The corresponding change in the Gibbs free energy (in the *T, P, N* ensemble as mentioned above) is estimated as:


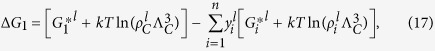


where 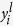 indicates the mole fractions of C conformers in the liquid phase equal to 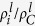. Hence, Equation (17) is re-arranged to the following form


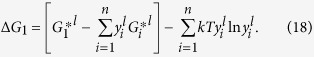


Secondly, we freeze the translational degrees of freedom of this conformer. The change in the Gibbs free energy in this case is estimated as:


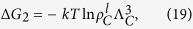


where 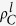 is the density of a conformer in the liquid phase.

At the third step, we remove continuum environment from over this conformer to put it into the gas phase. This leads to the following change in the Gibbs function:


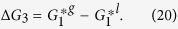


At the next step, we remove this conformer from its fixed positions in the gas phase. The corresponding change in the Gibbs free energy is estimated as:


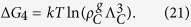


Finally, we release the constraint on this conformer and allow it to reach equilibrium among other (n − 1) conformers but with a new distribution of 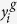 in the gas phase. The corresponding change in the Gibbs free energy is estimated as:


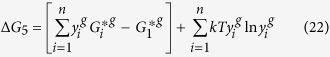


From combination of Equations (18, 19, 20, 21, 22) we have


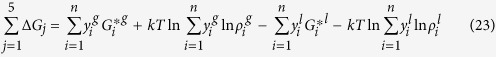


which can be simplified to


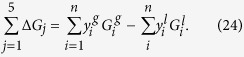


Since all conformers in the gas and liquid phases are in the equilibrium state,


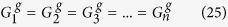



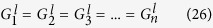


Therefore, IMD describes the driving forces in the evaporation process rather than translational contributions;


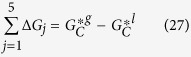


which can be applied to other complex molecules with multi-structural effects.

## Additional Information

**How to cite this article**: Nasiri, R. Revisiting kinetic boundary conditions at the surface of fuel droplet hydrocarbons: An atomistic computational fluid dynamics simulation. *Sci. Rep.* **6**, 25572; doi: 10.1038/srep25572 (2016).

## Figures and Tables

**Table 1:** Parameters^*^ obtained from fitting the data in Fig. 3.

Hydrocarbons	*n*-Heptane^**^		*n*-Nonane		*n*-Decane		*n*-Dodecane	
Parameters/interfacial conformer i	1	2	1	2	1	2	1	2
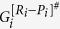	15.02	17.86	13.80	19.23	14.97	20.21	11.09	18.45
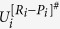	12.34	24.67	17.80	18.90	17.87	11.98	15.01	18.98
ω_i_	587	869	860	1075	596	987	421	1031

*Units are as follows: 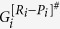 and 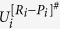 (kcal mol^−1^) and *ω*_i_ (cm^−1^).

**The parameters obtained at pressure 2 MPa.

## References

[b1] Milton J. R. Surfactants and interfacial phenomena (John Wiley & Sons, Inc., 2004).

[b2] Chen P. Molecular interfacial phenomena of polymers and biopolymers. CRC Press Boca Raton Boston (New York Washington, DC, 2005).

[b3] Constantinos G. V. Interfacial phenomena in electro-catalysis (Springer Science, 2011).

[b4] Clarence A. M. & Neogi P. In Interfacial phenomena equilibrium and dynamic effects (Surfactant Science Series Volume 139, New York) (CRC Press, Taylor & Francis Group, 2010).

[b5] Sheiko S. S. . Adsorption-induced scission of carbon–carbon bonds. Nature 440, 191–194 (2006). 16525468 10.1038/nature04576

[b6] Bolhuis P. G., Chandler D., Dellago C. & Geissler P. L. Transition Path Sampling: Throwing ropes over rough mountain passes, in the dark. Annu. Rev. Phys. Chem. 53, 291–318 (2002). 11972010 10.1146/annurev.physchem.53.082301.113146

[b7] Nagayama G. & Tsuruta T. A general expression for the condensation coefficient based on transition state theory and molecular dynamics simulation. J. Chem. Phys. 118, 1392–1399 (2003).

[b8] Ishiyama T., Yano T. & Fujikawa S. Kinetic Boundary Condition at a Vapour-Liquid Interface. Phys. Rev. Lett. 95, 084504 (2005). 16196864 10.1103/PhysRevLett.95.084504

[b9] Smith J. D., Cappa C. D., Drisdell W. S., Cohen R. C. & Saykally R. J. Raman Thermometry Measurements of Free Evaporation from Liquid Water Droplets. J. Am. Chem. Soc. 128, 12892–12898 (2006). 17002384 10.1021/ja063579v

[b10] Nasiri R., Gun’ko V. M. & Sazhin S. S. The Effects of Internal Molecular Dynamics on the Evaporation/Condensation of *n*-Dodecane. Theor. Chem. Acc. 134, 1–12 (2015).

[b11] Cao B.-Y., Xie J.-F. & Sazhin S. S. Molecular dynamics study on evaporation and condensation of n-dodecane at liquid–vapour phase equilibria J. Chem. Phys. 134, 164309 (2011). 21528962 10.1063/1.3579457

[b12] Chilukoti H. K., Kikugawa G. & Ohara T. A molecular dynamics study on transport properties and structure at the liquid–vapour interfaces of alkanes. Int. J. Heat Mass Trans. 59, 144–154 (2013).

[b13] Esenturk O. & Walker R. A. Surface vibrational structure at alkane liquid/vapour interfaces. J. Chem. Phys. 125, 174701 (2006). 17100455 10.1063/1.2356858

[b14] Xia T. K. & Landman U. Molecular evaporation and condensation of liquid *n*‐alkane films. J. Chem. Phys. 101, 2498–2507 (1994).

[b15] Fujikawa S. . Vapour-Liquid Interfaces, Bubbles and Droplets (Springer-Verlag, Heidelberg, 2011).

[b16] Winter A. Computational chemistry making a bad calculation. Nat. Chem. 7, 473–5 (2015). 25991524 10.1038/nchem.2267

[b17] NIST Chemistry WebBook, saturation Properties for *n*-heptane, *n*-nonane, *n*-decane, *n*-dodecane- temperature increments. Available at: http://webbook.nist.gov/chemistry/ (Accessed: 9^th^ August 2015).

[b18] Cummings P. C. . Phase transitions in nano-confined fluids: The evidence from simulation and theory. AIChE Journal. 56, 842–848 (2010).

[b19] Bureau L. Rate effects on layering of a confined linear alkane. Phys. Rev. Let. 99, 225503 (2007). 18233296 10.1103/PhysRevLett.99.225503

[b20] Skodje R. T. & Truhlar D. G. Parabolic tunnelling calculations. J. Phys. Chem. 85, 624–628 (1981).

[b21] Honnery D., Nguyen D. & Soria J. Microdroplet evaporation under increasing temperature conditions: experiments and modelling. Fuel 105, 247–257 (2013).

[b22] Ghasemi H., Wook B. S. & Sarwar khan Q. Experimental study on binary droplet evaporation at elevated pressures and temperatures. Combust. Sci. and Tech. 178, 1031–1053 (2006).

[b23] Ben-Naim A. Molecular theory of solutions (Oxford University Press Inc., New York, 2006).

